# Brucellosis, genital campylobacteriosis and other factors affecting calving rate of cattle in three states of Northern Nigeria

**DOI:** 10.1186/s12917-015-0317-9

**Published:** 2015-01-20

**Authors:** Hassan M Mai, Peter C Irons, Peter N Thompson

**Affiliations:** Department of Production Animal Studies, Faculty of Veterinary Science, University of Pretoria, Private Bag X04, Onderstepoort, 0110 South Africa; Animal Production Programme, School of Agriculture and Agricultural Technology, Abubakar Tafawa Balewa University, P. M. B. 0248, Bauchi, Nigeria

**Keywords:** Bovine genital campylobacteriosis, Brucellosis, Calving rate, Reproductive efficiency

## Abstract

**Background:**

Reproductive diseases limit the productivity of cattle worldwide and represent an important obstacle to profitable cattle enterprise. In this study, herd brucellosis and bovine genital campylobacteriosis (BGC) status, and demographic and management variables were determined and related to predicted calving rate (PrCR) of cattle herds in Adamawa, Kaduna and Kano states, Nigeria. Serum samples, preputial scrapings, questionnaire data, trans-rectal palpation and farm records were used from 271 herds. The Rose-Bengal plate test and competitive enzyme-linked immunosorbent assay were used for *Brucella* serology and culture and identification from preputial samples for BGC. A herd was classified as positive if one or more animals tested positive. The PrCR was determined as the number of calvings expected during the previous 6 and next 6 months as a percentage of the number of postpubertal heifers and cows in the herd. A multilevel linear regression model was used to estimate the herd-level effect of *Brucella abortus* seropositivity, *Campylobacter fetus* infection and other factors on calculated PrCR.

**Results:**

The reproductive performance of the cattle herds was generally poor: Only 6.5% of the nursing cows were pregnant and 51.1% were non-pregnant and acyclic; the mean annual PrCR was 51.4%. *Brucella abortus* and *C. fetus* infection of herds were independently associated with absolute reduction in PrCR of 14.9% and 8.4%, respectively. There was also a strong negative association between within-herd *Brucella* seroprevalence and PrCR. Presence of small ruminants, animal introduction without quarantine and the presence of handling facilities were associated with lower PrCR, whereas larger herd size, supplementary feeding, routine mineral supplementation and care during parturition were associated with higher PrCR.

**Conclusions:**

Brucellosis and BGC may be largely responsible for the poor reproductive performance of indigenous Nigerian cattle. Farmer education and measures to improve the fertility of cattle herds are suggested.

## Background

Cattle are the largest livestock enterprise in the agricultural sector in Nigeria, with a national herd of about 15.3 million [1]. However, the productivity and reproductive efficiency of indigenous Nigerian cattle are low [2,3]. About 95% of all food animal populations in Nigeria are in the hands of nomadic and semi-nomadic traditional farmers, who utilise relatively inefficient production systems [4]. Therefore, the causes of poor productivity need to be identified and addressed [5].

Reproductive indices reported in nomadic cattle herds in Nigeria include age at first calving of 60 months, calving interval of 17 to 24 months, annual calf crop of 40% and total lifetime number of calves produced by a cow of 2.5 [6]. Other reported indices include age at puberty of 40.2 months [7], calving to first conception of 7.8 months [8] and first service conception rate of 46.7% [9]. These indices are affected by several factors such as poor genetic material [2,3], adverse environmental factors [10], inadequate veterinary services [3], age and parity of the dam [5], inadequate nutrition [11], suckling [8], inadequate oestrus detection [12] and widespread infectious and parasitic diseases [3,13,14].

Measurement of annual calving percentage is a good measure of herd reproductive performance; however, it involves visiting the farm at least monthly for a period of one year to monitor and record calvings as they occur, and even then it depends on the farmer’s records, which are often poor and inadequate, or their recall. Predicted annual calving rate (PrCR), on the other hand, is a robust indicator of breeding performance and herd fertility, taking into account the number of pregnant animals and estimated ages of foetuses based on trans-rectal palpation, as well as estimated ages of calves in the herd at a single time point [5,15,16]. It is also independent of the season in which the data are collected, which can be a confounder when other indices are used in herds with seasonal calving patterns [17]. However, single-day examination of a herd and prediction of calving rate may be prone to bias in that it cannot account for future cases of abortion and is dependent on accurate aging of pregnancies.

Brucellosis, caused by *Brucella abortus*, and bovine genital campylobacteriosis, commonly caused by *Campylobacter fetus venerealis* [18], are known to be prevalent in Nigeria and have been implicated in infertility [13,14]. They result in huge economic losses due to abortion, repeat breeding, decrease in number of calves, culling and replacing affected animals and decreased milk production due to clinical mastitis [3,13,19-22]. In contrast, studies of trichomonosis in Nigeria have revealed a low or zero prevalence [23-25]. These venereal diseases are transmitted by communal bulls in management systems commonly found in various locations across Africa [26]; however, their influence on reproductive performance has not been well studied on a herd basis in communal farming systems [27,28].

The purpose of this study was firstly to estimate the reproductive efficiency of cattle herds in Northern Nigeria, as reflected by PrCR, and secondly to investigate the effect of brucellosis, BGC, and other managemental and environmental factors, on PrCR.

## Methods

This study was performed in conjunction with a survey to determine prevalence of and risk factors for brucellosis, BGC and trichomonosis in cattle herds of Northern Nigeria [23,29,30]. The research protocol was approved by the Animal Use and Care Committee and the Research Committee of the University of Pretoria (Protocol no. V073-08).

### Study areas and study design

**Figure 1:**
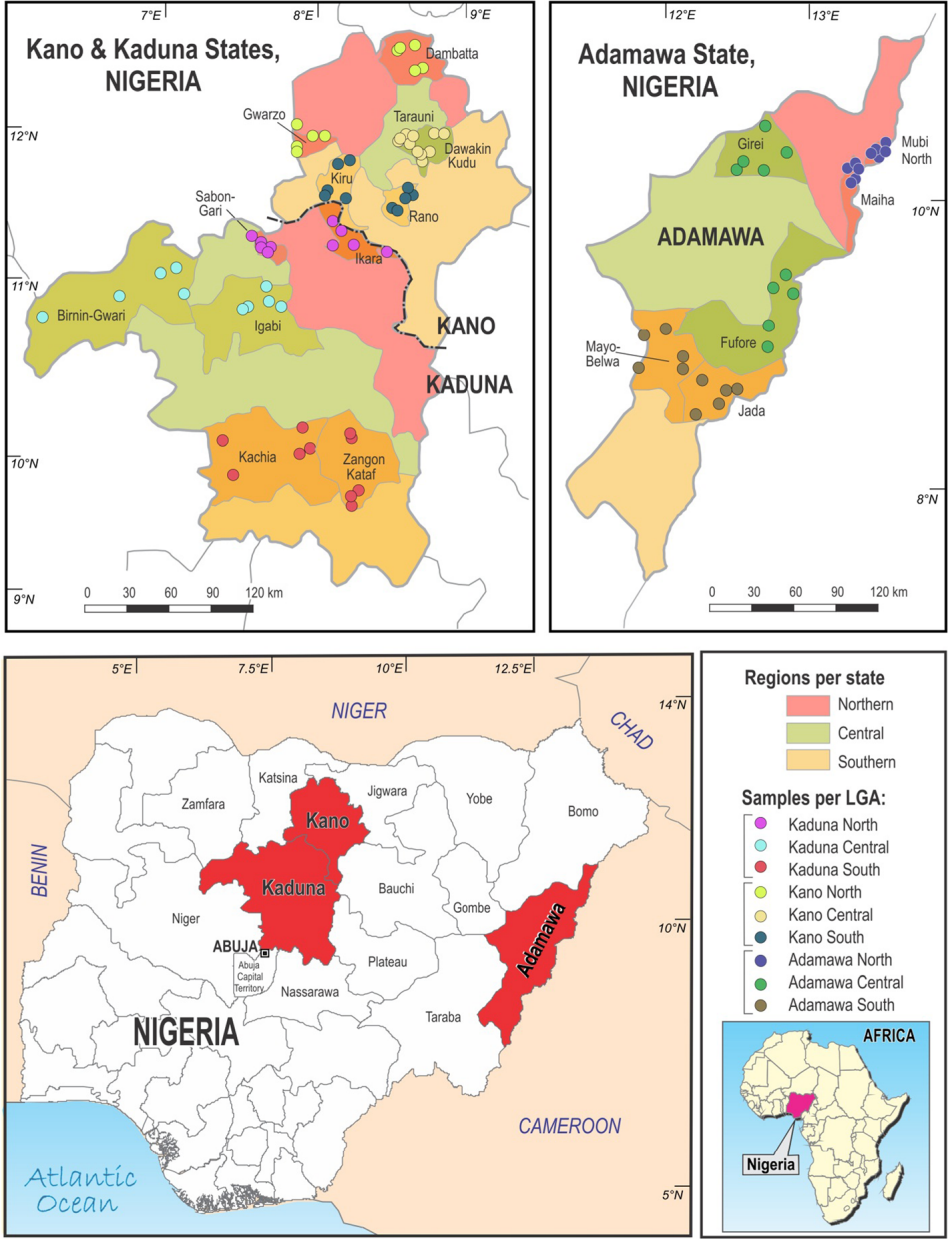
**Map of Nigeria showing the three States, 18 LGA’s and 89 wards sampled in Northern Nigeria.**

Three states, namely Adamawa, Kaduna and Kano, were selected from the 19 Northern states of Nigeria. Adamawa state is situated at 8-11°N and 11.5-13.5°E, Kaduna state at 9-11.3°N and 10.3-9.6°E, and Kano state is at 12°N and 9°E (Figure 1). All three states have Sudan or sub-Sudan savannah in the north and tropical grasslands of Guinea savannah in the south.

The study design was previously described [29]. Briefly, a cross sectional study was conducted using multistage cluster sampling. Sample size was calculated to estimate a 40% herd prevalence of brucellosis with 10% absolute precision and using a design effect of 2.8 to account for the multistage sampling design. Each of the three selected states was divided into three administrative geographical zones, and two local government areas (LGA’s) were randomly selected from each zone, giving a total of six LGA’s from each state, using as sampling frame a list of all LGA’s in each zone. Approximately 50% of wards were randomly selected from a list of all wards in each selected LGA (Figure 1). Since no sampling frames were available for selection of herds within wards, herds were selected by visiting the farms and enrolling them as they consented to participation. An average of three herds was selected per ward, giving an average of 15 herds selected per LGA. A total of 271 herds was sampled.

### Animal and herd classification

Selected herds were visited once each between July 2008 and June 2009. Herd and individual animal data collection, and animal sampling were done during this visit.

All the postpubertal bulls, postpubertal heifers, breeding bulls and cows were sampled in each selected herd. A postpubertal bull was defined as a bull that had been successfully mounting other cows or heifers by achieving intromission. A postpubertal heifer was a female that had been observed exhibiting oestrus or standing to be mounted by a bull or on trans-rectal examination had either of the functional structures, i.e. corpus luteum or follicle, on their ovaries.

Four management systems were encountered during the study. The pastoral management system was characterized by cattle grazing on fallow land close to the place of settlement of the owners during the rainy season but covering long distances, some even migrating, during the critical period of the dry season in search of natural pasture. Agro-pastoral management was characterized by cattle grazing locally and supplementation with mostly crop residues particularly during the dry and pre-rainy seasons. Commercial management systems were organized farms that were usually fenced with paddocked, improved pastures and concentrate provided as supplementary feeds. Zero-grazing systems were farms in which the cattle were confined or even tethered with restricted movement and feed was provided.

### Sample collection and testing for *Brucella abortus*

Animals selected for blood sampling for brucellosis were first calf heifers which had calved at least six weeks previously, cows and postpubertal heifers and bulls. About 10 ml of blood was collected from the jugular, coccygeal or saphenous veins into Vacutainer^®^ tubes, and placed into an ice bath and transported to the laboratory for centrifugation, serum separation and storage at -20°C until ready for analysis. The Rose-Bengal plate agglutination test (RBPT) for brucellosis using RBP antigen (VLA, Weybridge, UK) and confirmation of RBPT-positive samples with competitive enzyme-linked immunosorbent assay (c-ELISA) (VLA, Weybridge, UK) were carried out as recommended by OIE [31]. Sampling and testing methods are discussed in detail in Mai *et al.* [29], where the estimated animal-level sensitivity and specificity of the applied test system were calculated to be 87.9% and 99.8%, respectively.

### Sample collection and isolation of *Campylobacter fetus* from bulls

Preputial scrapings were collected from all breeding bulls and other postpubertal bulls in the herds as described by Irons *et al.* [32] and used to isolate *C. fetus* as described by OIE [31]. At 72 h, a representative of a dewdrop colony that was Gram-negative, vibroid in shape and oxidase- and catalase-positive was transferred to a blood agar base (Oxoid, CM0055), streaked for purity and incubated under microaerophilic conditions for 72 h. Each culture and incubation was verified by using control strains of *C. f. fetus* and *C. f. venerealis* (ATCC 33247 and 19438 respectively). These isolates obtained were subjected to biochemical testing for H_2_S production using TSI agar (Oxoid, CM0277B), aerobic growth, growth at 25°C and 42°C and in the presence of 1% glycine, 3.5% NaCl and sensitivity to cephalothin and nalidixic acid.

### Additional data collection

Interview-based, structured questionnaires were administered to the livestock owners on each farm at the time of sample collection, in order to gather information on potential animal-level and herd-level factors affecting PrCR. As far as possible, the herdsmen were interviewed in the presence of the owner or farm manager for about 30 to 45 minutes. Interview questions were focused on events on the farm over the past 12 to 24 months. Management, herd structure, location and environmental variables with a potential impact on PrCR were recorded. The reproductive status of each animal, such as suckling/non-suckling, age and parity, as well as method of breeding, feeding, breed, etc. were obtained.

Age was estimated using farm records, dentition and, in some cases, cornual rings. Body condition score (BCS) was obtained as described by Pullan [33] and assigned by the same veterinarian for all animals. Pregnancy diagnosis, including age of foetus, and cyclicity were determined in all mature females using trans-rectal palpation as described by Arthur *et al*. [34]. All data were stored in a Microsoft Excel spreadsheet (Microsoft Corp., Redmond, WA, U.S.A.).

### Determination of predicted annual calving rate

For the calculation of PrCR in each herd, the formula of Voh Jr and Otchere [5] and Stonaker *et al*. [15] was used to determine the number of animals likely to calve during a 12-month period (the previous 6 months and the next 6 months), as follows:

$$ \begin{array}{c}\mathrm{PrCR} = \mathrm{Number}\ \mathrm{of}\ \mathrm{calvings}\ \mathrm{due}\ \mathrm{in}\ \mathrm{one}\ \mathrm{year}/\mathrm{No}.\ \mathrm{of}\ \mathrm{postpubertal}\ \mathrm{heifers}\ \mathrm{and}\ \mathrm{cows}\\ {}=\left(b+e+g + 2h+i\right)/\left(a+b+c+d+e+f+g+h+i\right)\end{array} $$

where:

- *a* is the number of open, dry cows
- *b* is the number of open cows nursing a calf under 6 months of age
- *c* is the number of open cows nursing a calf 6 months of age and over
- *d* is the number of pregnant dry cows under 2 months of gestation
- *e* is the number of pregnant cows under 2 months of gestation and nursing a calf under 6 months of age
- *f* is the number of pregnant cows under 2 months of gestation and nursing a calf 6 months of age and over
- *g* is the number of pregnant dry cows at 2 months of gestation and over
- *h* is the number of pregnant cows at 2 months of gestation and over and nursing a calf under 6 months of age
- *i* is the number of pregnant cows at 2 months of gestation and over and nursing a calf 6 months of age and over.

The numerator for calculating annual PrCR therefore includes calves of 6 months of age or less (*b*, *e* and *h*) and all females which were pregnant on trans-rectal palpation, i.e. were more than 2 months in calf (*g*, *h* and *i*). This was considered the best period to choose as the pregnancy diagnosis results were accurate (carried out by an experienced veterinary surgeon and theriogenologist) and most farmers/herdsmen could remember calves of less than 6 months old [5,15,16]. The ‘*h*’ group was likely to produce two calves in one year and was therefore counted twice.

### Statistical analysis

The unit of analysis was the herd and the outcome variable was the PrCR. Each independent variable (brucellosis, BGC and the management and environmental variables) was tested for bivariable association with the outcome using Student’s *t*-test or ANOVA. Variables associated with the outcome at *P* < 0.2 were selected for the multivariable model. A multilevel, mixed-effects linear regression model with state as a fixed effect and nested random effects for LGA and ward was then constructed. Backward elimination was applied until all remaining variables were significant (*P* < 0.05), after which all other predictor variables were tested by adding them back into the model and retained if significant. Significance of the random effects for LGA and ward was assessed by comparing models with and without random effects using a likelihood ratio test. Fit of the final model was evaluated using a plot of residuals versus fitted values and a normal probability plot of residuals. The association between within-herd *Brucella* seroprevalence and PrCR was also determined. All statistical analyses were done using STATA 12 (Stata Corporation, College Station, TX, USA) and a significance level of *α* = 0.05 was used.

## Results

### Herd structure

The structure of the 271 herds sampled is shown in Table 1. The average bull: female ratio was one mature male to eight mature females. The herd size ranged between 7 and 119 animals (median: 34; interquartile range (IQR): 25, 43).

**Table 1:** **Herd structure, breed, management system and reproductive status of cattle sampled from three states of Northern Nigeria**

**Variables and categories**	**Total**	**Proportion of group (%)**
Herd structure		
Bulls	602	6.0
Heifers	1,134	11.3
Cows	3,068	30.4
Bull calves and growers	1,285	12.8
Young bulls	1,038	10.3
Heifer calves and growers	1,276	12.7
Young heifers	1,663	16.5
Total^a^	10,066	
Breed		
Bunaji	3,097	64.4
Gudali	870	18.1
Other *Bos indicus*	448	9.3
*Bos taurus*	120	2.5
*B. taurus* x *B. indicus*	272	5.7
Total^b^	4,807	
Management system		
Pastoral	1,263	26.3
Agro-pastoral	2,793	58.1
Commercial	650	13.5
Zero-grazing	101	2.1
Total^b^	4,807	
Reproductive status		
Suckling	1,818	43.3
Non-pregnant	1,545	36.8
Cyclic	609	14.5
Non-cyclic	936	22.3
Pregnant	273	6.5
Non-Suckling	2,384	56.7
Non-pregnant	1,290	30.7
Pregnant	1,094	26.0
Total^c^	4,202	

^a^Total number of animals in the sampled herds.

^b^Number of mature animals.

^c^Number of mature females.

### Reproductive parameters

Because a few herds had no postpubertal heifers or cows, PrCR could be calculated for only 251 herds. The mean annual PrCR was 51.4%, ranging between 0% and 100%, while the pregnancy rate, defined as the proportion of cows and postpubertal heifers that were pregnant, was 32.5%.

### Reproductive status and BCS

A total of 4,202 females consisting of 1,134 heifers and 3,068 cows were studied. The proportion suckling, and pregnancy and cyclicity status are shown in Table 1. The BCS ranged from 2 to 5 (median: 3; IQR: 3, 4). Using two categories of BCS (≤3 and ≥3.5), there was a significant difference in the BCS between cyclic and non-cyclic cows (*P* < 0.0001) and between suckling and non-suckling cows (*P* < 0.0001) (data not shown).

### Reproductive status of heifers and parity of cows and heifers

The reproductive performance records of heifers indicated that at <2 years some heifers started cycling; peak cyclicity (55%) and pregnancy (57%) were attained at 3 and 5 years respectively. The median age at puberty was between 2 and 3 years (Table 2). Table 3 shows the distribution of parity by age. The median age at first calving was between 4 and 5 years.

**Table 2:** **Reproductive status of heifers sampled from the three states of Northern Nigeria**

**Age (years)**	**Cyclic**	**Acyclic or reproductive problem**	**Pregnant**	**Total**
< 2	2	5	0	7
2	21 (21.2)	65 (65.7)	13 (13.1)	99
3	208 (54.6)	92 (23.1)	81 (21.3)	381
4	212 (44.2)	54 (11.3)	214 (44.6)	480
5	54 (36.2)	10 (6.7)	85 (57.0)	149
6	1	10	2	13
7	0	5	0	5
Total	498	241	395	1134

**Table 3:** **Age and parity of cattle sampled from three states of Northern Nigeria**

**Age**	**Parity**											
**(years)**	**0**	**1**	**2**	**3**	**4**	**5**	**6**	**7**	**8**	**9**	**10**	**Total**
<2	7	0	0	0	0	0	0	0	0	0	0	7
2	99	4	0	0	0	0	0	0	0	0	0	103
3	381	46	0	0	0	0	0	0	0	0	0	427
4	480	241	21	0	0	0	0	0	0	0	0	742
5	149	581	131	40	2	0	0	0	0	0	0	903
6	13	284	285	65	21	0	0	0	0	0	0	668
7	5	50	204	107	25	6	0	0	0	0	0	397
8	0	6	91	143	57	10	2	0	0	0	0	309
9	0	2	6	78	50	22	5	1	0	0	0	164
10	0	0	6	33	50	36	7	1	0	0	0	133
11	0	0	1	8	14	13	8	6	1	0	0	51
12	0	0	0	6	7	15	9	10	6	1	0	54
13	0	0	0	0	0	2	3	3	1	1	0	10
14	0	0	0	0	1	0	0	0	0	0	1	2
15	0	0	0	0	0	0	0	0	0	1	3	4
Total	1134	1214	745	480	227	104	34	21	8	3	4	3974
% of total	28.5	30.5	18.7	12.1	5.7	2.6	0.9	0.5	0.2	0.1	0.1	

### Number of calves per cow lifetime in the herd and productive life of the cows

A total of 2,840 cows were examined for which we had complete information about their ages (Table 3). The cows had produced a total of 6,054 calves, i.e. 2.1 calves produced/cow. Furthermore, Table 3 shows that very few animals were kept beyond 10 years.

### Factors associated with PrCR

**Figure 2:**
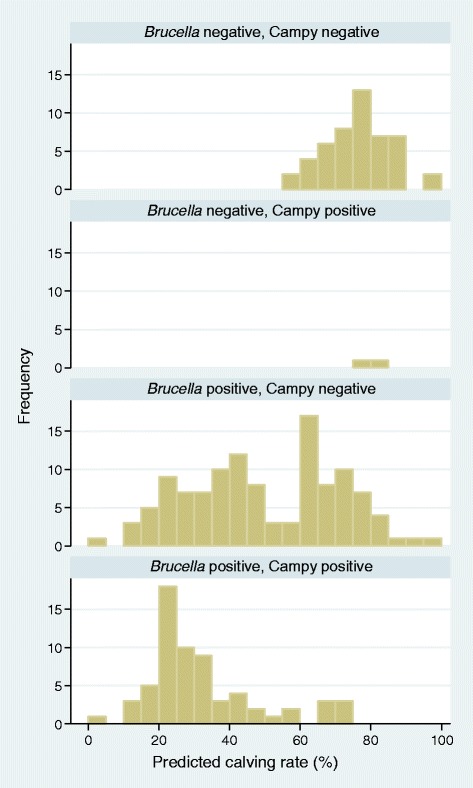
**Predicted calving rate in** ***Brucella abortus*** **positive and negative herds, and** ***Campylobacter fetus*** **positive and negative herds in three states of Northern Nigeria.**

**Figure 3:**
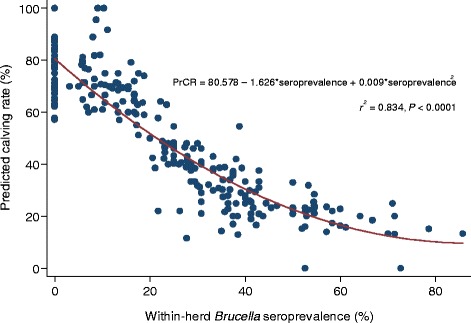
**Scatter plot of predicted calving rate (PrCR) vs. within-herd** ***Brucella*** **seroprevalence, with least squares quadratic fit, in cattle sampled from three states of Northern Nigeria.**

The distribution of the various environmental and managemental factors and their bivariable association with PrCR at the herd level are shown in Table 4. The crude absolute difference in PrCR between *Brucella* positive and *Brucella* negative herds was 33.2%, while that between *C. fetus* positive and *C. fetus* negative herds was 24.2%. All of the 59 herds that were *Brucella* negative had a PrCR of over 50%, while 124/192 (65%) of the *Brucella* positive herds had a PrCR of <50% (Figure 2). The mean PrCR for *Brucella* positive, *Brucella* negative, *C. fetus* positive and *C. fetus* negative herds were 43.6%, 76.8%, 33.1% and 57.3% respectively. In addition, there was a strong negative association between within-herd *Brucella* seroprevalence and PrCR (*P* < 0.001) (Figure 3).

**Table 4:** **Bivariable analysis of categorical predictors for predicted calving rate in herds in three states of Northern Nigeria**

**Predictor and level**	**No. tested**	**Calving rate (%)**		***P*** **-value**
		**Mean**	**SD**	
*Brucella* infection^a^				<0.001
No	59	76.8	9.2	
Yes	192	43.6	21.8	
*Campylobacter fetus* infection^a^				<0.001
No	166	57.3	22.2	
Yes	66	33.1	18.0	
State^a^				0.033
Adamawa	87	46.1	23.5	
Kaduna	98	55.2	22.8	
Kano	66	52.7	25.8	
Method of breeding^a^				0.026
AI and natural mating	44	52.5	24.0	
AI only	11	70.1	25.0	
Natural mating only	196	50.1	23.8	
Use of AI^a^				0.11
No	196	50.1	23.8	
Yes	55	56.0	25.0	
Management system^a^				<0.001
Zero-grazing	3	76.2	12.3	
Commercial	26	66.2	25.4	
Agro-pastoral	146	58.1	21.3	
Pastoral	76	32.6	17.3	
Supplementary feeding^a^				<0.001
None	25	21.9	7.6	
Fodder/bran	105	46.3	22.1	
Concentrate	121	62.0	21.5	
Mineral supplementation^a^				<0.001
No	69	32.2	17.6	
Yes	182	58.7	22.2	
Pasture establishment^a^				0.122
No	187	50.0	23.8	
Yes	64	55.5	24.9	
Water source^a^				<0.001
Piped	69	63.2	21.2	
Natural flowing	112	46.6	22.5	
Natural static	70	47.6	25.8	
Housing^a^				<0.001
Open barbed wire	153	46.4	23.3	
Open half way and roofed	66	63.1	24.6	
Open solid enclosure	32	51.5	18.9	
Hygiene/floor type^a^				<0.001
Floored	63	63.1	23.0	
Unfloored/natural bear earth	188	47.5	23.3	
Isolation and observation of the cow during parturition and removal of afterbirth^a^				<0.001
No	94	35.1	19.1	
Yes	154	61.4	21.4	
Regular herd prophylactic measures^a^				<0.001
No	97	40.0	22.1	
Yes	154	58.6	22.5	
Borrow/share bull^a^				<0.001
No	166	60.4	21.8	
Yes	85	33.9	18.2	
Presence of small ruminants^a^				<0.001
No	97	65.6	19.5	
Yes	154	42.5	22.5	
Presence of dogs^a^				0.036
No	227	52.9	24.5	
Yes	24	37.5	13.7	
Presence of chickens^a^				0.0002
No	161	55.7	23.3	
Yes	90	43.8	23.9	
Multiple herds^a^				0.013
No	166	54.1	23.6	
Yes	85	46.1	25.3	
Purpose of keeping animals^a^				0.0002
Small scale local dairy	187	52.5	24.1	
Dairy and Beef	29	61.3	23.2	
Beef	35	37.5	18.9	
Initial purchase of stock from a market^a^				<0.001
Inherited	118	55.1	24.0	
Other farms	14	71.5	21.2	
Market	119	45.4	27.7	
Buying-in new animals and quarantine^a^				<0.001
Buy <3 + quarantine	30	68.4	11.8	
Buy >3 or no quarantine	147	38.3	20.2	
Close herd	74	70.6	15.9	
Socio-economic status of farmer^a^				0.031
Full-time	176	53.6	23.7	
Part-time	75	46.4	24.9	
Specialist attending to animals^a^				<0.001
No	48	32.6	17.9	
Yes	203	55.9	23.3	
Presence of crush/local chute or other means of handling/restrain at the farm^a^				0.061
No	187	49.8	23.9	
Yes	64	56.3	24.9	

^a^Variable significant (*P* < 0.20) for calving rate and therefore considered in the multivariable model.

The final regression model of factors associated with PrCR is shown in Table 5. The random effects for LGA and ward were not significant and therefore the normal multiple regression model without random effects was used. The residuals were normally distributed and the residual vs. fitted plot showed no evidence of non-linearity or heteroscedasticity. After adjustment for confounding by the other variables in the model, *Brucella* herd infection was associated with an absolute reduction in PrCR of 14.9%. In addition to this, *C. fetus* herd infection was associated with a further reduction in PrCR of 8.4%.

**Table 5:** **Factors associated with predicted calving rate in cattle herds in Northern Nigeria: results of a multiple linear regression model**

**Risk factor and level**	**Coefficient**	**95% CI**	***P*** **- value**
*Brucella* infection			
No	1	-	-
Yes	-14.9	-20.01, -9.62	<0.001
*Campylobacter fetus* infection			
No			
Yes	-8.41	-12.93, -3.88	<0.001
State			
Adamawa	1	-	-
Kaduna	1.76	-2.84, 6.35	0.452
Kano	-0.24	-5.40, 4.92	0.928
Supplementary feeding			
None	1	-	-
Fodder and bran	6.54	0.46, 12.63	0.044
Concentrate	7.86	0.46, 15.30	0.037
Mineral supplementation			
No	1		
Yes	6.45	1.71, 11.20	0.008
Isolation and observation of cow during parturition and removal of afterbirth			
No	1		
Yes	7.54	3.09, 11.98	0.001
Small ruminants			
No	1	-	-
Yes	-7.81	-12.41, -3.22	0.001
Buy in new animals			
Closed herd	1	-	-
Buy <3 + quarantine	-6.44	-12.53, -0.38	0.038
Buy >3 or no quarantine	-15.23	-20.31, -10.16	<0.001
Presence of crush, chute or other form of restraint on the farm			
No	1	-	-
Yes	-9.97	-16.08, -3.76	0.002
Herd size			
≤ 15	1	-	
> 15	4.98	1.17, 8.80	0.011

Herds that gave fodder and bran were associated with 6.5% higher PrCR (*P =* 0.044) and herds that gave concentrate with 7.9% higher PrCR (*P =* 0.037) than herds that did not. In addition, mineral supplementation and isolation and observation of cows during parturition and removal of afterbirth were associated with higher PrCR than herds in which these practices were absent. Furthermore, the presence of small ruminants, the presence of a handling facility and the introduction of new animals, particularly the introduction of >3 animals without quarantine, were significantly associated with lower PrCR in such herds (Table 5). Herd size was initially not significant in the bivariable analysis but after adding it to the final model and adjusting for other variables there was a significant positive association with PrCR.

## Discussion

Reproductive indices are vital in the determination and management of herd fertility. It is apparent from this study that several factors are responsible for poor reproductive efficiency of cattle in Northern Nigeria. Previous studies on the reproductive performance of cattle in traditional herds in Northern Nigeria are more than two decades old [5] and there is a lack of data quantifying the impact of infectious causes of infertility [13,14]. This report provides current information on reproductive efficiency and factors affecting calving rates in cattle in Nigeria. It is the only report that considers various management systems in one study.

The average herd size of 37 in agro-pastoral production systems obtained in this study is similar to 38.3 reported by Voh Jr and Otchere [5] in agro-pastoral herds; but the herd size of 34.1 in pastoral herds (data not shown) is lower than 45.9 reported by Otchere [35] in the same management system.

From the global perspective, the previous few decades have witnessed a steady rise in bovine infertility [36]. The overall calving rate of 51.4% found in this study is similar to the 52 to 55% calving rate reported in Colombia [15] and the 55% observed by Voh Jr and Otchere [5] in the traditional agro-pastoral system in Nigeria. The pregnancy rate of 32.5% in this study is lower than the 42% reported by Voh Jr and Otchere [5]. Nevertheless, our study does not provide conclusive evidence to support a decline in fertility of the study population in Northern Nigeria.

It is apparent from this study that brucellosis and BGC have a significant impact on PrCR, and that there is a clear negative relationship between within-herd *Brucella* seroprevalence and PrCR. The outcome of brucellosis such as abortion, retained afterbirth, stillbirth and birth of weak calves or calf mortality affect the overall calving rate of infected herds. This tends to agree with reports by Aguair *et al*. [37] and Degefa *et al*. [38]. It is also consistent with the report that a 10% decrease in the number of calves was observed in *Brucella* positive cows [19]. Bovine genital campylobacteriosis causes similar clinical signs and therefore may be associated with infertility thereby lowering calving rate and other reproductive indices [39,40]. Due to the fact that almost all *C. fetus* positive herds were also positive for *Brucella*, it was not possible to accurately quantify the impact of BGC alone. However, a combination of brucellosis and BGC was associated with poorer PrCR in this study than brucellosis alone (Figure 2), which would suggest that BGC has an additional negative effect. Despite this, our data confirm that it is possible to maintain good calving rate with only brucellosis or BGC infections, and even with both infections present a PrCR in excess of 70% is possible, provided that the within-herd seroprevalence of brucellosis is below about 20% (Figure 3). The fact that females often abort once and following that they reproduce normally in the case of brucellosis, and the acquired immunity conferred by *C. fetus* challenge, may explain the acceptable PrCR observed in some infected herds.

Although management system was not significant in the multivariable analysis of PrCR, the model showed that the observed difference in PrCR between the management systems was partially accounted for by the other variables in the multivariable model. In the bivariable analysis, the PrCR differed significantly between the various management systems (*P* < 0.001). The crude PrCR being lowest in the pastoral system may be as a result of the movement of the pastoral Fulani herdsmen and interaction of their cattle with other Fulani herdsmen particularly at watering points during the dry season which may expose them to infection thereby lowering the PrCR. In previously published data from the same study we showed that the presence of brucellosis was positively associated with the pastoral management system [29].

It was shown from this study that providing supplementary feeding and mineral supplementation were associated with higher PrCR, as were the isolation and observation of cows during parturition and removal of the afterbirth, and the presence of a handling facility were associated with lower PrCR. Such effects may be by proxy, in that the education level of the herd owner, availability of other sources of income, focus on other activities may all have impact on the general level of management, condition and health of the herd. Likewise, larger herd size is likely to be associated with increased animal movements, with the associated increased risk of contact with infectious agents. Indeed, farmers that introduced > 3 animals without quarantine were found to have 15% lower PrCR than farmers that did not. In the initial crude analysis, the association with herd size was obscured due to confounding; in the multivariable analysis PrCR was significantly associated with herd size, with larger herds having higher PrCR. The reason for this is not clear. The commercial and zero-grazing herds showed higher PrCR but had smaller herd sizes.

It was observed that over 61% of the multiple herd owners introduced >3 animals without quarantine in their herds. This is a risky practice due to the potential for introducing infections that may lower the calving rate. Reports indicate that ownership of multiple herds potentially increases the risk of a herd being infected with brucellosis [41], which may also affect the calving rate.

Herds that had small ruminants had significantly lower PrCR. Cross infection of infectious reproductive diseases may be possible between species thereby lowering the PrCR. This tends to agree with findings by Megersa *et al*. [19] regarding mixed herds/flocks. The association between presence of a handling facility and lower PrCR may be due to the fact that such farmers may be likely to share their facilities with other farmers, leading to increased contact with other herds.

The median age at puberty observed in this study (2 to 3 years) is shorter than reports by Mukasa-Mugerwa [3] who showed average age at puberty of *Bos indicus* as 40 months. However, the median age at first calving agreed with estimates of 4 to 5 years reported by Voh Jr and Otchere [5] and 5 years by Zemjanis [6]. In addition, the reported age at first calving in indigenous tropical cattle of between 3 and 5 years, between 4 and 7 years for the second time and between 5 and 8 years for the third [42] are consistent with our findings. This study also revealed that age at first calving in cattle in Northern Nigeria can also be as low as 2 to 3 years, meaning that some animals attained puberty and conceived at about 1 to 2 years old. Oyedipe *et al*. [11] indicated that under improved management where seasonal nutritional stress is reduced, it is possible to achieve average age at first calving a little over 3 years.

The previously reported reproductive lifespan of cattle in Northern Nigeria of up to 10 years [5] is consistent with our findings. Almost all of the cows had been culled by the age of 10 years. We can therefore conclude from this finding that the productive life of cattle in this study area is up to 10 years. The low lifetime number of calves per cow may be attributed to late age at first calving, long calving intervals and early culling age. It is an underestimation of true lifetime production in that it includes animals which are still in the productive state. Suckling and nutrition are in a large part responsible for this reproductive inefficiency [5].

Body condition score is a management tool that has proved useful in the assessment of the nutritional status of dairy and beef cows [43,44]. Poor BCS of cows, mainly caused by poor management, was also considered to play a major role in reducing pregnancy rates [45]; their results further suggest that an abrupt loss of nutritional status postpartum can impair uterine involution, and cause pregnancy failure in the early foetal development period when the placentomes develop. In addition, a one unit reduction in BCS from previous partum to 30 days postpartum resulted in a 2.4-fold increase in pregnancy loss [45]. Highly significant associations between BCS and pregnancy status (*P* < 0.0001) and BCS and cyclicity status (*P* < 0.0001) were observed in this study.

The limitations to this study were that the determination of age at puberty and age at first calving relied on observations of the farmers and herdsmen who are mostly uneducated, and on the herd size, the management system, etc. These may introduce some bias to the study.

## Conclusion

The reproductive performance of the cattle herds studied in Northern Nigeria was generally poor. *Brucella abortus* and *C. fetus* infections were associated with reduced PrCR. In addition, presence of small ruminants, lack of quarantine and presence of handling facility were also associated with lower PrCR. Suckling and nutrition contributed to the high prevalence of anoestrus. However, improved feeding, attention during parturition and herd size improved the PrCR. Herd health management programmes, proper feeding and care during parturition should be encouraged while failure to quarantine, sharing handling facilities and mixing herds with small ruminants should be avoided.

## Abbreviations

BCS: Body condition score
BGC: Bovine genital campylobacteriosis
c-ELISA: Competitive enzyme-linked immunosorbent assay
IQR: Interquartile range
LGA: Local government area
RBPT: Rose-Bengal plate agglutination test
PrCR: Predicted calving rate

## References

[1] Anon: Animal population. World Animal Health Information Database (WAHID). 2011.

[2] Pullan NB (1979). Productivity of white Fulani cattle in Jos Plateau, Nigeria. II. Nutritional factors. Trop. Anim. Health Prod..

[3] Mukasa-Mugerwa E: A review of reproductive performance of female *Bos indicus* (Zebu) Cattle. ILCA Monograph No.6. 1989.

[4] Rikin UM (1988). Brucellosis of cattle in Nigeria. Proposals for a control under intensive and extensive husbandry systems. Acta Vet. Scand..

[5] Voh AA, Otchere EO (1989). Reproductive performance of Zebu cattle under traditional agro-pastoral management in Northern Nigeria. Anim. Reprod. Sci..

[6] Zemjanis R: Veterinarians and animal production in Nigeria. A Paper presented at the Annual meeting of the Nigerian Veterinary Medical Association. Nigeria: Port-Harcourt; 25-28^th^ September, 1974.

[7] Knudson PM, Sohael AS (1970). The Vom herd: a study of the performance of a mixed Friesian/Zebu herd in a tropical environment. Trop Agric (Trinidad).

[8] Eduvie LO, Dawuda PM (1986). Effect of suckling on reproductive activities of Bunaji cows during the postpartum period. J Agric Sci Cambridge.

[9] Voh AA, Buvanendran V, Oyedipe EO (1987). Artificial insemination of indigenous Nigerian cattle following synchronization of oestrus with PGF2& 1. Preliminary fertility trial. Brit Vet J.

[10] Mai HM (1997). Some environmental and physiological factors affecting fertility rates in artificially inseminated cattle herds. MSc thesis.

[11] Oyedipe EO, Osori DIK, Akerejola O, Saror D (1982). Effects of levels of nutrition on onset of puberty and conception rates of Zebu heifers. Theriogenology.

[12] Mai HM, Ogwu D, Eduvie LO, Voh AA (2002). Detection of oestrus in Bunaji cows under field conditions. Trop. Anim. Health Prod..

[13] Mshelia GD, Amin JD, Egwu GO, Yavari CA, Murray RD, Woldehiwet Z (2010). Detection of antibodies specific to *Campylobacter fetus* subsp. *venerealis* in the vaginal mucous of Nigerian breeding cows. Vet. Ital..

[14] Ocholi RA, Kwaga JKP, Ajogi I, Bale JO (2004). Phenotypic characterization of *Brucella* strains isolated from livestock in Nigeria. Vet. Microbiol..

[15] Stonaker HH, Villar J, Osorio G, Salazar J (1976). Differences among cattle and farms as related to beef cow reproduction in the Eastern plains of Colombia. Trop. Anim. Health Prod..

[16] Reed JBH, Doxey DL, Forbes AB, Finlay RS, Geering IW, Smith SD (1974). Productive performance of cattle in Botswana. Trop. Anim. Health Prod..

[17] Fahey J, O’Sullivan K, Crilly J, Mee JF (2002). The effect of feeding and management practices on calving rate in dairy herds. Anim. Reprod. Sci..

[18] Schmidt T, Venter EH, Picard JA (2010). Evaluation of PCR assays for the detection of *Campylobacter fetus* and identification of subspecies in South African. J. S. Afr. Vet. Assoc..

[19] Megersa B, Biffa D, Abunna F, Regassa A, Godfroid J, Skjerve E (2011). Seroprevalence of brucellosis and its contribution to abortion in cattle, camel, and goat kept under pastoral management in Borana, Ethiopia. Trop. Anim. Health Prod..

[20] Esuruoso GO, Geemwy WA (1979). Current status of brucellosis in Nigeria and preliminary evaluation of probable cost and benefit of proposed brucellosis control programme for the country. Proceedings of the Second International Symposium on Veterinary Epidemiology and Economics.

[21] Akhtar A, Riemann HP, Thurmond MC, Franti CE (1993). The association between antibody titres against *Campylobacter fetus* and milk production efficiency in dairy cattle. Vet. Res. Commun..

[22] Bawa EK, Adekeye JO, Oyedipe EO, Umoh JU (1991). Prevalence of bovine campylobacteriosis in indigenous cattle of three states in Nigeria. Trop. Anim. Health Prod..

[23] Mai HM, Irons PC, Kabir J, Thompson PN (2013). Prevalence of genital campylobacteriosis and trichomonosis of bulls in Northern Nigeria. Acta Vet. Scand..

[24] Adeyeye AA, Ate IU, Bale JO, Lawal AI (2011). A survey for bovine trichomoniasis in cattle at slaughter in the Sokoto metropolitan abattoir, Sokoto state, Nigeria. Sokoto J Vet Sci.

[25] Akinboade OA (1980). Incidence of Bovine Trichomoniasis in Nigeria. Rev. Elev. Med. Vet. Pays Trop..

[26] Tekleye B, Kasali OB, Mukawa-Mugerwa E, Scholtens RG, Yigzaw T: Infertility problems of cattle in Africa. Proceedings of the 6^th^ Tanzania Veterinary Association Scientific Conference. Arusha: Tanzania; 5^th^-7^th^ December, 1988:152.

[27] Njiro SM, Kidanemariam AG, Tsotetsi AM, Katsande TC, Mnisi M, Lubisi BA (2011). A study of some infectious causes of reproductive disorders in cattle owned by resource-poor farmers in Gauteng Province, South Africa. J. S. Afr. Vet. Assoc..

[28] Mokantla E, McCrindle CME, Sebei JP, Owen R (2004). An investigation in to the causes of low calving percentage in communally grazed cattle in Jericho, North West Province. J. S. Afr. Vet. Assoc..

[29] Mai HM, Irons PC, Kabir J, Thompson PN (2012). A large seroprevalence survey of brucellosis in cattle herds under diverse production systems in Northern Nigeria. BMC Vet. Res..

[30] Mai HM, Irons PC, Kabir J, Thompson PN (2013). Herd-level risk factors for *Campylobacter fetus* infection, *Brucella* seropositivity and within-herd seroprevalence of brucellosis in cattle in Northern Nigeria. Prev. Vet. Med..

[31] OIE (2011). Manual of Diagnostic Tests and Vaccines for Terrestrial Animals.

[32] Irons PC, Schutte AP, Van Der Walt ML, Bishop GC, Coetzer JAW, Thompson G, Tustin RC (2004). Genital Campylobacteriosis in cattle. Infectious Diseases of Livestock.

[33] Pullan NB (1978). Condition scoring of white Fulani cattle. Trop. Anim. Health Prod..

[34] Arthur GH, Noakes DE, Pearson H, Parkinson TJ (1996). Veterinary Reproduction and Obstetrics.

[35] Otchere EO, von Kaufmann R, Chater S, Blench R (1986). Traditional Cattle Production in the Subhumid Zone of Nigeria. Livestock Systems Research in Nigeria’s Subhumid Zone.

[36] Lopez-Gatius F (2003). Is fertility declining in dairy cattle? A retrospective study in northeastern Spain. Theriogenology.

[37] Aguiar DM, Cavalcante GT, Labruna MB, Vasconcellos SA, Rodrigues AAR, Morais ZM (2007). Risk factors and seroprevalence of *Brucella* spp. in cattle from western Amazon, Brazil. Arq Inst Biol.

[38] Degefa T, Duressa A, Duguma R (2011). Brucellosis and some reproductive problems of indigenous Arsi cattle in selected Arsi zones of Oromia regional state, Ethiopia. Global Vet.

[39] Campero CM, Anderson ML, Walker RL, Blanchard PC, BarBano L, Chiu P (2005). Immunohistochemical identification of *Campylobacter fetus* in natural cases of bovine and ovine abortions. J. Vet. Med. B.

[40] Jimenez DF, Perez AM, Carpenter TE, Martinez A (2011). Factors associated with infection by *Campylobacter fetus* in beef herds in the Province of Buenos Aires, Argentina. Prev. Vet. Med..

[41] Richey EJ, Harrell CD: *Brucella abortus* disease (brucellosis) in beef cattle. IFAS Extension: University of Florida; 1997:1-6. http://www.agro.uba.ar/users/catala/Informacion%20Brucelosis/brucellosis%201.pdf.

[42] Wilson RT (1985). Livestock production in central Mali: Reproductive aspects of sedentary cows. Anim. Reprod. Sci..

[43] Hady PJ, Demecq JJ, Kaneene JB (1994). Frequency and precision of body condition scoring in dairy cattle. J. Dairy Sci..

[44] Montiel F, Ahuja C (2005). Body condition and suckling as factors influencing the duration of postpartum anestrus in cattle: a review. Anim. Reprod. Sci..

[45] Lopez-Gatius F, Santolaria P, Yaniz J, Rutllant J, Lopez-Bejar M (2002). Factors affecting pregnancy loss from gestation day 38 to 90 in lactating dairy cows from a single herd. Theriogenology.

